# Is the olfactory system of cartilaginous fishes a vomeronasal system?

**DOI:** 10.3389/fnana.2013.00037

**Published:** 2013-10-17

**Authors:** Sara Ferrando, Lorenzo Gallus

**Affiliations:** Department of Earth, Environmental and Life Science (DISTAV), University of GenoaGenoa, Italy

**Keywords:** Chondrichthyes, olfactory receptors, vomeronasal receptors type 2, evolution of the olfactory system, fish olfactory system

In vertebrates, two chemosensory systems are devoted to olfaction: the main olfactory system (MOS) and the vomeronasal system (VNS). In tetrapods, the olfactory and VNSs are actually two distinct sensory epithelia lining two distinct organs, projecting to the olfactory bulb (OB) and the accessory olfactory bulb (AOB), respectively (Mombaerts, 2004). In fish, there is only one “olfactory” epithelium, showing the morphological and molecular features of both MOS and VNS (Eisthen, 2004; Ubeda-Bañon et al., 2011) and projecting to the OB, that is somehow regionalized according to the taxon (Hansen et al., 2003; Hansen and Zielinski, 2005). An actual AOB has been recently described in lungfish (González et al., 2010), suggesting that this structure is present in all Sarcopterygians.

**Figure 1:**
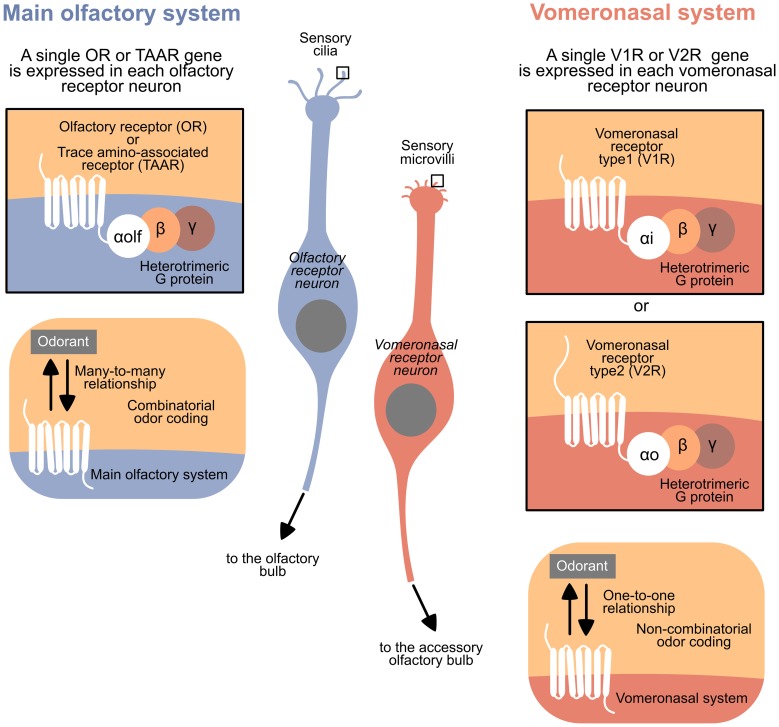
**Summary of the general differences between the main olfactory system and the vomeronasal system in vertebrates**.

The VNS and MOS present peculiar features in all vertebrates, facilitating the recognition of these systems in classes where they are not anatomically distinct. The typical olfactory receptor neurons (RNs) bear cilia as dendritic specializations and express a receptor from the largest family of genes in vertebrate, the Olfactory Receptor family (ORs), or from the Trace Amine-Associated Receptor family (TAARs). Moreover, each receptor family has a defined G protein alpha subunit; specifically, ORs and TAARs, expressed in ciliary RNs, are coupled to the subunit Gαolf. However, typical vomeronasal RNs bear microvilli as dendritic specializations and express a receptor from the family Vomeronasal Receptors 1 (V1Rs) or Vomeronasal Receptors 2 (V2Rs). The V1Rs are typically coupled to the subunit Gαi, and V2Rs are coupled to Gαo (and to Gαq in fish) (Berghard and Buck, 1996; Jia and Halpern, 1996; Hansen et al., 2004; Hansen and Zielinski, 2005; Liberles and Buck, 2006; Hashiguchi and Nishida, 2007; Spehr and Munger, 2009) (see Figure 1 for a summary).

Some exceptions to the expression of ORs and V1Rs in the MOS and VNS, respectively, have been previously reported: some ORs have been detected in the VNS of mice (Lévai et al., 2006), while V1Rs have been described in the MOS of *Xenopus*, goats and humans (Rodriguez et al., 2000; Wakabayashi et al., 2007; Date-Ito et al., 2008).

Previous studies concerning the number of receptors involved in vertebrate chemosensitivity have shown that the ORs range from 11 genes in teleost species (with a peculiar low number in *Tetraodon nigroviridis*) to 154 genes in *Danio rerio*, reaching hundreds of genes in amphibians and mammals (Niimura, 2009). The TAARs are generally smaller in number, ranging from 13 to 109 genes in teleost species and from 5 to 22 genes in mammals (Hashiguchi and Nishida, 2007). The vomeronasal gene families are even smaller as the V1Rs are not represented in fish, and only 5 to 6 genes have been identified in teleosts, 21 genes have been identified in frogs and 187 genes have been identified in mice (Shi and Zhang, 2007; Hashiguchi et al., 2008); the V2Rs in teleosts range from 11 to 46 genes, while 249 have been identified in frogs, and less than 100 genes have been identified in mammals (Shi and Zhang, 2007; Hashiguchi et al., 2008).

The hypotheses that the VNS and MOS can detect, respectively, pheromones and odors or innately recognizable and learned odors have often been questioned (e.g., Kelliher, 2007), although many authors have proposed a clear difference in the tuning of olfactory and vomeronasal receptors (e.g., Grus and Zhang, 2008; Spehr and Munger, 2009). There is ample evidence for the “different tuning hypothesis”; this hypothesis relies on the different evolutionary patterns observed in the receptor families. The differential tuning hypothesis predicts that MOS receptors typically detect an overlapping set of ligands and are more likely to be evolutionarily conserved over time than specialist VNS receptors, which would evolve in a more lineage-specific manner. Indeed, the functional profile of the VNS chemoreceptor repertoire evolves much faster than that of the MOS chemoreceptor repertoire (Grus and Zhang, 2008). Moreover, the ORs bind different ligands with different affinities, consistent with the combinatorial nature of olfactory sensitivity (Malnic et al., 1999). The vomeronasal receptors are similar to other G protein-coupled receptors (GPCRs), showing a strong affinity for a particular ligand (Grus and Zhang, 2008).

The MOS has been identified in all the classes of vertebrates, while the VNS is lacking in some groups, such as birds and primates, which presumably have lost this system (Ubeda-Bañon et al., 2011). In agnate species, remarkably, the primordial elements of the VNS could already be present, although, for example, the V2Rs are completely absent (Libants et al., 2009; Ubeda-Bañon et al., 2011).

Here, we proposed that in Chondrichthyes, unique in vertebrates, the sense of smell could primarily (or completely) rely on the VNS. Chondrichthyes are one of seven vertebrate classes, and this class is further divided into two subclasses, Elasmobranchs and Holocephali. In the phylogenetic tree, these subclasses are located before the split between Sarcopterygians (from which tetrapods arose) and Actinopterygians, although these subclasses are members of jawed vertebrates. The peripheral olfactory organs of these vertebrates are large olfactory rosettes, with a huge sensory surface (Theisen et al., 1986; Meredith and Kajiura, 2010; Howard et al., 2013). The ultrastructure of the sensory epithelium has been studied in different species, highlighting the absence of ciliated RNs, a unique condition in all studied fishes and, notably, in all studied vertebrates (Holl, 1973; Bronshtein, 1976; Theisen et al., 1986; Takami et al., 1994). Unfortunately, only the genome of the Chondrichthye species elephant shark *Callorhinchus milii* (subclass Holocephali) has been sequenced to date (Venkatesh et al., 2007). The analysis of the *C. milii* genome revealed the presence of only 1 functional gene belonging to ORs and 2 genes for TAARs (Hussain et al., 2009; Niimura, 2009). This result is consistent with the absence of ciliated RNs in the sensory epithelium of Chondrichthyes, as reported above. However, 1 gene belonging to V1Rs (this receptor family is not typically numerous in aquatic vertebrates) and 32 genes belonging to V2Rs have been identified in *C. milii* (Grus and Zhang, 2009). The immunohistochemical detection of G protein α subunits in the olfactory organ of *Scyliorhinus canicula* (Elasmobranch) and *Chimaera monstrosa* (Holocephali) did not reveal the presence of Gαolf, highlighting the presence of Gαo in virtually all RNs, consistent with the presence of V2Rs (Ferrando et al., 2009, 2010).

Thus, the ultrastructural, genomics and immunohistochemical data are consistent with the hypothesis that the olfactory system of Chondrichtyes primarily relies on V2Rs, which are coupled to Gαo and expressed in microvillus RNs, as in other vertebrates. Recent physiological studies of the chemo-sensitivity in two elasmobranch species demonstrated that these organisms are able to detect amino acids and bile salts (Meredith et al., 2012). In teleost, bile salts are recognized as odorants through ciliated Gαolf- and ORs-expressing RNs (Døving et al., 2011). However, we cannot rule out that V2Rs in Chondrichthyes also recognize bile salts as ligands.

Other data, obtained from many years of research concerning Chondrichthyes, might support our hypothesis concerning the existence of an entire class of vertebrates without a real MOS. For example, the ultrastructural study of the OB of the elasmobranch *Sphyrna tiburo* provided evidence for a particular type of synapse, the perforate synapse. Dryer and Graziadei (1996) commented that perforated synapses “*have been described in the rat medial amygdaloid nucleus (a vomeronasal nerve projection area) and in the accessory olfactory bulb, but none has been described so far in the main olfactory bulb*.”

Although other data are required to demonstrate the strong reduction (or absence) of the MOS in Chondrichthyes, the evidence reported here supports this possibility. The notable difference from other vertebrates does not reflect a primitive condition as lampreys, which diverged from the vertebrate tree before Chondrichthyes, have ~30 ORs genes in the genome and Gαolf-immunoreactive ciliated RNs in the olfactory epithelium (Frontini et al., 2003; Laframboise et al., 2007; Niimura, 2009). Rather, the reduction (or absence) of MOS in Chondrichthyes should be ascribed to a peculiar evolutionary path of this class.

A class of organisms in which the entire sense of smell relies on a family of receptors (the V2Rs) with a strong affinity for the ligand, unlike the ORs family, could not use combinatorial olfactory coding, as normally observed in vertebrates (Malnic et al., 1999). The combinatorial code depends on the different affinities of different ORs for different odorants, resulting in a wide variety of detected odorants and a different perceived odor according to the odorant concentrations. Instead, the chemosensory system of Chondrichthyes could be concentration independent above a given threshold. A small number of detected odorants, detected by a single receptor instead of a combination of receptors in a concentration-independent manner, could generate a chemosensory system less disturbed by the environmental mixture of chemicals. A recent study demonstrated that elasmobranchs and teleost fishes have comparable amino acid thresholds of detection (Meredith and Kajiura, 2010), but the detection of a single olfactory cue among many other cues should also be considered in future studies.

The odor coding in Chondrichthyes, different from that of all other vertebrates, is further supported by the recent discovery of the somatotopic organization of the OB in elasmobranchs, a unique organization among other vertebrates, in which the OB shows chemotopic organization (Meredith et al., 2013).

Notably, the ORs, TAARs, and V1Rs gene families, belonging to the rhodopsin-like GPCRs, are present in both Agnatha and Gnathostomes, while the V2Rs gene family, belonging to metabotropic Glutamate Receptor-like receptors, is present only in Gnathostomes (Suárez et al., 2012). Thus, V2Rs were likely a relatively new family of chemosensory genes, when Chondrichthyes diverged from the vertebrate phylogenetic tree. Thus, the full exploitation of these new receptors could have provided Chondrichthyes with an advantage in predatory activity. However, the predominance of VNS in the sense of smell could reflect the need to compensate for an impairment of the MOS, providing evidence for an as yet unknown element during the evolution of this class.

Thus, a demonstration of the lack of MOS in Chondrichthyes or the discovery of MOS molecules in this class is needed to obtain insight into the biology of these fascinating fishes and the evolution of MOS and VNS in vertebrates.
